# Protocol for a randomised controlled trial of treatment of asymptomatic candidiasis for the prevention of preterm birth [ACTRN12610000607077]

**DOI:** 10.1186/1471-2393-11-19

**Published:** 2011-03-11

**Authors:** Christine L Roberts, Jonathan M Morris, Kristen R Rickard, Warwick B Giles, Judy M Simpson, George Kotsiou, Jennifer R Bowen

**Affiliations:** 1 Clinical and Population Perinatal Health Research, Kolling Institute of Medical Research, University of Sydney, NSW Australia; 2 Department of Obstetrics and Gynaecology, University of Sydney, NSW Australia; 3 Department of Obstetrics and Gynaecology, Royal North Shore Hospital, NSW Australia; 4 School of Public Health, University of Sydney, NSW Australia; 5 Department of Microbiology and Infectious Diseases, Royal North Shore Hospital, NSW Australia; 6 Department of Neonatology, Royal North Shore Hospital, NSW Australia

## Abstract

**Background:**

Prevention of preterm birth remains one of the most important challenges in maternity care. We propose a randomised trial with: a simple *Candida *testing protocol that can be easily incorporated into usual antenatal care; a simple, well accepted, treatment intervention; and assessment of outcomes from validated, routinely-collected, computerised databases.

**Methods/Design:**

Using a prospective, randomised, open-label, blinded-endpoint (PROBE) study design, we aim to evaluate whether treating women with asymptomatic vaginal candidiasis early in pregnancy is effective in preventing spontaneous preterm birth. Pregnant women presenting for antenatal care <20 weeks gestation with singleton pregnancies are eligible for inclusion. The intervention is a 6-day course of clotrimazole vaginal pessaries (100 mg) and the primary outcome is spontaneous preterm birth <37 weeks gestation.

The study protocol draws on the usual antenatal care schedule, has been pilot-tested and the intervention involves only a minor modification of current practice. Women who agree to participate will self-collect a vaginal swab and those who are culture positive for Candida will be randomised (central, telephone) to open-label treatment or usual care (screening result is not revealed, no treatment, routine antenatal care). Outcomes will be obtained from population databases.

A sample size of 3,208 women with *Candida *colonisation (1,604 per arm) is required to detect a 40% reduction in the spontaneous preterm birth rate among women with asymptomatic candidiasis from 5.0% in the control group to 3.0% in women treated with clotrimazole (significance 0.05, power 0.8). Analyses will be by intention to treat.

**Discussion:**

For our hypothesis, a placebo-controlled trial had major disadvantages: a placebo arm would not represent current clinical practice; knowledge of vaginal colonisation with *Candida *may change participants' behaviour; and a placebo with an alcohol preservative may have an independent affect on vaginal flora. These disadvantages can be overcome by the PROBE study design.

This trial will provide definitive evidence on whether screening for and treating asymptomatic candidiasis in pregnancy significantly reduces the rate of spontaneous preterm birth. If it can be demonstrated that treating asymptomatic candidiasis reduces preterm births this will change current practice and would directly impact the management of every pregnant woman.

**Trial registration:**

Australian New Zealand Clinical Trials Registry ACTRN12610000607077

## Background

### Preterm birth - burden of disease

Preterm birth (birth before 37 weeks of gestational age) is the leading cause of perinatal morbidity and mortality worldwide [1]. Although improvements in neonatal care have increased survival for preterm infants, prevention research and knowledge to date have not managed to reduce the rate of premature birth [1,2]. The frequency of preterm birth ranges from 5% to 13% in high income counties and the incidence is increasing [3,4]. Approximately half the preterm births are due to spontaneous preterm birth, for which there are no known effective prevention measures [3].

Preterm birth is associated with admission to neonatal intensive care, severe morbidity in the first weeks of life, prolonged hospital stay after birth, and readmission to hospital in the first year of life [1]. It is implicated in at least two-thirds of infant deaths and nearly half of long-term neurologic disability [1]. Surviving infants, especially those born before 32 weeks gestation, have substantially increased risks of chronic health disorders; neurodevelopmental impairments (including cerebral palsy, mental retardation, visual and auditory deficits); difficulties with motor skills, speaking, writing, mathematics, behaviour, and physical education; dysfunction in other cognitive areas, such as attention, visual processing, academic progress, and executive function; low educational achievements and employment; and greater needs for services such as physician visits, occupational or physical therapy, nursing or medical procedures [1]. Over the first 10 years of life hospital inpatient admissions, inpatient days and costs respectively are 130%, 77%, and 443% higher for children born very preterm than for children born at term [5]. Even late preterm birth (34 -37 weeks) is associated with increased risks of infant death, cerebral palsy and social isolation [6]. Clearly, prematurity and its associated sequelae have an enormous negative psychosocial and emotional effect on the family.

### Infection and preterm birth

Microbiological studies suggest that intrauterine infections account for 25-40% of preterm births; but this is likely to be an under-estimate because intrauterine infection is difficult to detect with conventional culture techniques [3]. The mechanisms by which intrauterine infections lead to preterm labour relate to activation of the innate immune system [7]. Microorganisms are recognised by pattern-recognition receptors which in turn elicit the release of inflammatory chemokines and cytokines [3,7,8]. Microbial endotoxins and proinflammatory cytokines stimulate the production of prostaglandins, other inflammatory mediators, and matrix-degrading enzymes [3,7,8]. Prostaglandins stimulate uterine contractility and initiate labour, while degradation of extracellular matrix in the fetal membranes leads to preterm prelabour rupture of the membranes [3,7,8].

Recognition that it is ascending infection that leads to preterm birth has led to a number of studies that have evaluated the treatment of vaginal infection in pregnancy to reduce preterm birth [9-13]. To date, most of the intervention trials have found little effect on the rate of preterm birth. Antibiotics prior to pregnancy do not reduce preterm birth [9]. A Cochrane systematic review of the effectiveness of antibiotics for the treatment of bacterial vaginosis to prevent prematurity concludes that there is "little evidence that screening and treating all pregnant women with asymptomatic bacterial vaginosis will prevent preterm birth and its consequences" [12]. Similarly, antibiotic treatment of vaginal *Ureaplasma urealyticum *and *Chlamydia trachomatis *have not significantly reduced preterm birth rates [10,13]. Finally, there is a possibility that the use of metronidazole to treat trichomoniasis in pregnancy is associated with an increased risk of prematurity [11].

In contrast to these negative results, a recent randomised controlled trial of antenatal screening and treatment for bacterial vaginosis, candidiasis and/or trichomoniasis in early pregnancy (15-19 weeks gestation) among over 4 000 asymptomatic pregnant women reduced the spontaneous preterm birth rate by 46% [14]. The benefit appeared to be limited to those women who were treated for asymptomatic candidiasis, however this interpretation is based on post-hoc subgroup analysis of this trial. On the other hand, two observational studies that included *Candida *in pregnancy, conducted in low socioeconomic populations in the USA in the 1980 s [15,16], found no association with preterm birth although one found a significantly increased risk of intra-uterine growth retardation among women with candidiasis (aOR 1.94, 95%CI 1.2-3.1) [16]. Importantly, in the latter, studies screening was performed later in pregnancy (22-30 weeks gestation)[15,16] and did not include women with low-level *Candida *colonisation [15]. Such conflicting results emphasise the need to evaluate interventions with appropriately designed randomised clinical trials.

### Candidiasis

Candidiasis commonly called "yeast infection" or "thrush" is a fungal infection (mycosis) which occurs when there is overgrowth of any of the *Candida *species, of which *Candida albicans *is the most common [17]. *Candida *is normally found on skin or mucous membranes including the vagina [17]. However, if the environment becomes imbalanced, such as when the normal acidity of the mucous membrane changes or when the hormonal balance changes, *Candida *can multiply [17]. When that happens, symptomatic candidiasis can occur, ranging from oral thrush and vaginitis, to systemic and potentially life-threatening diseases. *Candida *infections of the latter category are usually confined to severely immunocompromised persons.

Vaginal candidiasis, usually (90%) due to *Candida albicans*, is often harmless and causes no symptoms [18,19]. However it can also cause vaginal soreness, itching and discharge [18,20]. Approximately 75% of all healthy women of fertile age suffer from at least one episode of vaginal candidiasis, with associated physical and psychological morbidity and approximately half will have a recurrence [18,21]. Vaginal candidiasis (referred to as 'candidiasis' in this protocol) is not traditionally classified as a sexually transmitted infection, since it occurs in celibate women, and is considered part of the normal vaginal flora [22]. This does not mean that sexual transmission does not occur, although the contribution of sexual transmission to the pathogenesis of infection is unknown [20]. Importantly treatment of male partners has not been effective in reducing recurrence of vaginal candidiasis [23].

Up to 40% of pregnant women may have vaginal colonisation by *Candida *species, a two-fold increase from the prevalence rate in non-pregnant women [14-16,24-26]. This association is believed to be driven by increased levels of circulating oestrogens and deposition of glycogen and other substrates in the vagina during pregnancy [20,27].

### Candidiasis and preterm birth

The role of *Candida *colonisation on the pathway to preterm birth has not been pursued with the vigour ascribed to bacterial vaginosis and other vaginal organisms, possibly because it is considered a normal vaginal commensal organism. However, several factors indicate an association between vaginal candidiasis and preterm birth:

• the rate of spontaneous preterm birth in women with untreated, asymptomatic candidiasis is higher than among women with normal flora - 7.6% versus 2.8% (P = 0.009) in a low-risk obstetric population [14],

• in a high-risk population, there was increased rate of preterm birth among women with non-albicans *Candida *compared with uncolonised women (15.4% versus 11.3%, P = 0.05)[15]

• there is a concordance in risk factors for *Candida *colonisation and risk factors for preterm birth including African-American women, maternal medical conditions, low socio-economic status, and bacterial vaginosis [14,15]

• *Candida *can be isolated from the amniotic fluid of women with spontaneous preterm birth [28,29]

• within the oral endothelium *Candida *increases gene expression of metalloproteinases [30]. Similar effects are seen in vitro in the genital tract as *Candida *increases metalloproteinase 9 production by chorioamniotic membranes [31]. Metalloproteinase 9 is a connective tissue remodelling protein which has an important role in the origin of preterm labour and preterm premature rupture of the membranes.

Furthermore, there is emerging evidence that eradication of Candida in pregnancy may reduce the risk of preterm birth and late miscarriage:

• Kiss and colleagues reported that screening and treatment for infections between 15 and 19 weeks of gestation in 4429 asymptomatic women reduced the preterm birth rate by 46% [14]. Those with: candidiasis (n = 586, 14.1%) were treated with vaginal clotrimazole; trichomonas with vaginal metronidazole; and bacterial vaginosis with vaginal clindamycin. The benefit appeared to be in women treated for candidiasis: spontaneous preterm birth occurred in only 8 of 289 (2.8%) women treated for candidiasis compared with 22 of 291 (7.6%) women with untreated candidiasis in the control group (OR 0.35, 95% CI 0.14-0.84 P = 0.009) [27]. However this is a post-hoc analysis and the numbers are small.

• Three reports based on population-based data (Hungarian Case-Control Surveillance of Congenital Abnormalities) in over 38,000 women have also demonstrated that preterm birth was reduced in those women who were treated with clotrimazole in pregnancy [32-34]. These results were obtained after extensive adjustment for confounding variables, exclusion of significant recall bias, and despite a 65% excess of urinary tract infections (which predispose to preterm birth) in the group treated with clotrimazole. A competing explanation is that pregnancy outcomes were better in health conscious women who sought treatment with clotrimazole. However, this would be expected to apply also to the women in this cohort who were treated with metronidazole, whose rate of preterm births was unchanged. The authors speculated that the protective effect of clotrimazole for preterm birth may be attributable to the restoration of the normal colonisation of the female genital tract and its known antibacterial and/or antiprotozoal effect. Whilst these studies involved many women and support a benefit from treating *Candida*, they are observational rather than randomized trials [27].

• Finally, in a retrospective study of 1230 pregnancies among low socioeconomic status Latina women in New York, the 371 women treated with intravaginal azoles for *Candida *vaginitis were significantly less likely to deliver preterm than the 859 women without vaginitis (relative risk 0.51, P = 0.009)[35]

### Treatment of *Candida *in pregnancy

There are no randomised placebo-controlled trials of clotrimazole in pregnancy to reduce preterm birth. A Cochrane Systematic review, including 10 trials (1046 women), of treatment for thrush in pregnancy found:[36] (i) topical treatment to be effective in eradicating candidiasis, (ii) topical imidazoles (such as clotrimazole) to be more effective than nystatin, and that (iii) a treatment regimen of 6 or 7 days is superior to those of shorter duration. Specifically vaginal clotrimazole for 6 days reduced *Candida *colonisation in pregnancy to 12% at 3-6 weeks post treatment compared with 58% of women receiving placebo (OR 0.14, 95%CI 0.06-0.31) [37]. Two trials involving 81 pregnant women show treatment lasting 7 days was more effective (98% eradication) than treatment for 4 days (49% eradication, OR 0.09, 95% CI 0.03-0.24) [38,39]. However, the endpoint of these trials has been the successful eradication of *Candida *and importantly no pregnancy outcomes were reported. A randomised controlled trial of clotrimazole is necessary to provide the best evidence of whether treatment of asymptomatic *Candida *colonisation reduces preterm delivery.

Local application of clotrimazole vaginal pessaries or cream is generally well tolerated [36]. Occasionally skin reactions (burning, stinging, or redness) can occur. Clotrimazole has been used by a large number of pregnant women and women of childbearing age without any proven increase in the frequency of malformations or other direct or indirect harmful effects on the fetus having been observed [40]. A large population-based database study did not demonstrate risks to the fetus following exposure to clotrimazole in pregnancy [41]. Furthermore, the susceptibility of both *Candida albicans *and non-albicans *Candida *vaginal isolates to azole antifungal agents such as clotrimazole supports the continued practice of azole antifungal agents for empirical therapy of uncomplicated *Candida *vaginitis [42].

### Pilot study of clotrimazole to prevent birth in women with *Candida*

The methods for the proposed trial have been pilot tested in an open-label trial of clotrimazole versus usual care for women with asymptomatic candidiasis [43]. The pilot study informed the development of the full trial protocol and achievement of competitive funding. The results are reported in detail elsewhere, but briefly the participation rate was 64%; the rate of *Candida *colonisation in asymptomatic women was 19.8%; women were not inconvenienced by participation, laboratory testing and medication dispensing were problem-free; the follow-up rate was 99% and there was a trend to a reduction in spontaneous preterm birth in the clotrimazole group. Study procedures, including self-collection of specimens and treatment administration were well accepted by participants.

## Methods/Design

### Aim

to conduct a randomised controlled trial to answer the clinical question

In women with asymptomatic vaginal candidiasis early in pregnancy does treatment with clotrimazole prevent spontaneous preterm birth (less than 37 weeks gestation)?

### Hypothesis

that treatment of asymptomatic vaginal candidiasis with clotrimazole is associated with a 40% reduction in spontaneous preterm birth <37 weeks gestation.

### Study Design

We will use a prospective, randomised, open-label, blinded-endpoint (PROBE) study design with 2 arms [44,45]:

#### Treatment

6-day treatment with vaginal clotrimazole

#### Usual care

screening result is not revealed, no treatment, routine antenatal care

Although, double-blind, placebo-controlled randomised trials are regarded as the 'gold standard' of clinical trials, they do have some disadvantages. For our hypothesis, a placebo arm would not represent current clinical practice (no screening and no treatment). Knowledge of vaginal colonisation with *Candida *may change participants behaviour such that they seek active therapy (clotrimazole vaginal preparations are available over the counter in Australia) rather than, or in addition to, the study medication [45]. Further, the time, expense and feasibility of obtaining a placebo preparation should not be under-estimated [46]. And finally, as a vaginally administered placebo will necessarily contain an alcohol preservative, it may be biologically active and the possibility of an independent affect on vaginal flora cannot be excluded.

These disadvantages can be overcome by a PROBE study design [44,45]. As in our pilot study, the PROBE study design maintains strict randomisation and allocation concealment procedures but asymptomatic women will be randomised to open-label treatment (Canestan^®^), or no treatment and the screening result is not revealed (consistent with current clinical practice) [43-45]. The primary endpoint (preterm birth) is unambiguously defined and those assessing the end-point will be blinded to the screening results and treatment allocation. Another advantage of using open-labelled medication is that patient management adheres more closely to routine clinical practice than in double-blind, placebo-controlled trials, making the results more generalisable to the pragmatic management of patients [44].

### Setting

The study will be carried out in at least five participating maternity hospitals. A range of antenatal care options are provided in these hospitals including standard antenatal clinic care, midwifery-led care, birth centre care, GP-shared care and private obstetric care.

### Participants/Eligibility criteria

Pregnant women presenting for antenatal care prior to 20 weeks gestation with singleton pregnancies will be eligible for inclusion into the study. Treatment by 20 weeks of gestation is likely to be necessary to prevent preterm birth. *Exclusions*: women who present beyond 20 weeks gestation or have a history of hypersensitivity to clotrimazole will be ineligible. Women with symptomatic vaginal infection due to *Candida *will be treated and are therefore ineligible for the study.

### Sample size

A sample size of 3,208 women with *Candida *colonisation (1,604 per arm of the trial) is required to detect a 40% reduction in spontaneous preterm births (compared with term birth) among women with asymptomatic candidiasis from 5.0% in the control group to 3.0% for those women treated with clotrimazole (significance 0.05, power 0.8). This is a more conservative risk reduction than that found in the trial by Kiss [14], where analysis of this subgroup suggested treatment of asymptomatic candidiasis was associated with a 65% reduction in spontaneous preterm birth but the numbers were small and the subgroup analysis was post-hoc.

Recruitment of 3,208 women will require 16,040 women to be screened based on a *Candida *carriage rate of 20%. In our pilot study the *Candida *carriage rate was 19.6% and the participation rate among eligible women was 64%. If a participation rate of 64% could be maintained across all participating centres, ~25,000 would need to be approached to achieve screening of 16,040 women. A lower participation rate means more women would need to be approached. We expect that a participation rate of at least 60% could be maintained across all centres and this would mean approaching ~26,000 women.

### Recruitment and collection of baseline data

The study procedure is feasible as it draws on usual antenatal care in pregnancy, and is similar to the Kiss trial (see Table 1). The research nurse will ask eligible women to participate, explain the trial and obtain informed consent, collect baseline data and ask women to self-collect a vaginal swab. This is only a minor departure from current practice.

**Table 1:** Trial Schema

		Timing (data collection)
Identification & screening of eligible women (N = 16,040)*		Routine antenatal visit at 12-19 weeks (baseline data, *Candida *testing)
**↓**		
Randomisation of women with asymptomatic *Candida *(n = 3208)		< 1 week after *Candida *testing
**Clotrimazole** **↓**	**Usual care** **↓**	
Follow-up	Follow-up	28-32 weeks gestation (questionnaire)
**↓**	**↓**	
Birth	Birth	Primary and secondary outcomes collected from obstetric database

* primary outcome data (preterm birth rates) will be obtained for all women screened regardless of the result

As women of child-bearing age are known to be very mobile [47], participants will be asked to provide their parents and other alternative contact details (eg friend or relative) to enhance subsequent follow-up. Women will also be asked to specify their preferred method of contact - women in our pilot study have shown a strong preference for email contact. Self-collection of vaginal swabs is commonly used for screening in pregnancy [48]. We have also successfully used this method of specimen collection in a study of vaginal *Ureaplasma urealyticum *colonisation in pregnancy[49] and in our pilot study [43]. The detection rate of *Candida *using self-collection is not different from techniques that are more invasive, requiring the use of a speculum [50].

### Randomisation

Eligible women who are culture positive for *Candida *will be randomised to receive active treatment with clotrimazole or usual care. The randomisation schedule will be prepared and centrally administered by a researcher not involved in patient care. A computer random number generator will be used to prepare the randomisation schedule with permuted blocks and 1:1 randomisation. The women randomised to open-label clotrimazole treatment will be notified by phone by a research nurse. A central pharmacy will be responsible for dispensing the study medication which will be sent to participants by mail from the coordinating centre. This approach has been acceptable and successful in the pilot study.

### Specimen collection and transport

Surveillance for *Candida *isolates will be conducted at 12-19 weeks of pregnancy. A vaginal swab will be self-collected and inoculated immediately into Amies Transport Media, stored at room temperature and transported to Royal North Shore Hospital (Sydney) within 24 hours. All swabs will be identified with date of collection, patient's study number, packaged according to IATA guidelines, and transported to Pacific Laboratory Medicine Service (PaLMs), Royal North Shore Hospital.

### Isolation and identification of *Candida *from vaginal swabs

The detection and identification of *Candida *from vaginal swabs is readily achieved. The swab will be inoculated onto full-plates of chromogenic agar (CHROMagar™Candida, CHROMagar, Paris, France) to isolate, provisionally speciate and semi-quantitatively enumerate (scant, moderate, or heavy) *Candida *species and to obtain colonies for further work such as susceptibility testing by broth microdilution methods. This medium is chosen to ensure detection of mixed infections. Cultures will be incubated for 72 hours at 35°C in 5% CO_2_. Identification to species level will be based on colony morphology, and colour.

### Intervention

The treatment group will receive a 6-day course of commercially available vaginal clotrimazole pessaries (Canestan^®^). Women will be advised to insert one pessary as gently and deeply as possible into the vagina while lying on her back, preferably at night, for 6 nights. We have chosen 6 days of treatment because this is: supported by the Cochrane Systematic Review of treatment for *Candida *eradication in pregnancy [36], this is how commercially available vaginal clotrimazole is marketed[40] and this was the regimen used in the Kiss trial [14]. Women who miss one or more daily doses will be advised to insert a single pessary as soon as they remember or the next evening, and continue using the pessaries until the course of treatment is finished.

### Follow-up

A brief (< 5 minutes) follow-up questionnaire will collect information about symptoms of candidiasis, use of antibiotics and/or corticosteroids (risk factors for symptomatic candidiasis), treatment of candidiasis (including study medication, doctor prescribed and over-the-counter medications), treatment side-effects and compliance with the treatment regimen. The questionnaire will be web-based with email notification or mailed with a reply-paid envelope according to the participants stated preference at trial entry. These secondary outcomes will be collected at 28-32 weeks, a gestation when the potential for unmasking the 'no treatment' arm will be irrelevant.

Care of all women and their babies will be otherwise managed according to the standard practices of the model of maternity care she has chosen. Women who develop symptoms of *Candida *infection will be offered appropriate treatment. The results of the vaginal swab culture taken at the time of trial entry will not be disclosed nor be available in the pregnancy record.

### Outcomes and Explanatory Factors

#### Baseline data

Brief baseline data will be collected to assess comparability of the study groups. The baseline assessment will include age, socio-demographic data, smoking and alcohol use, medical conditions, history of candidiasis, history of prior pregnancies including preterm birth, gestational age at enrolment and method of gestational age assessment (ultrasound or dates). This information will be obtained from existing databases (as outlined below) or where this is not available from the participants at study entry.

##### Primary Outcome

birth <37 weeks gestation following spontaneous onset of labour or following preterm prelabour rupture of membranes.

The primary and secondary outcomes will be obtained (with informed consent) from existing computerised obstetric and hospital databases. This is a novel and cost-efficient approach [51]. We have previously demonstrated that the outcomes for this study are reliably collected in routinely collected obstetric and hospital databases [52-56]. It also allows identification of transfers to another hospital. The latter are uncommon and we will utilise health record linkage to determine outcomes for these few women who would otherwise be lost to follow-up. Consequently, we anticipate negligible loss to follow-up in this trial. Because this approach is novel, we will validate the primary outcome (preterm birth) by reviewing the medical records of all women identified in the computerised databases as delivering preterm, those where gestational age is missing (usually <1%) and a random sample of those recorded as term deliveries. We estimate that approximately 300 records will need to be reviewed to validate preterm birth.

##### Secondary outcomes

Include any preterm birth <37 weeks, medically indicated preterm birth <37 weeks (including indication), preterm birth <32 weeks, preterm prelabour rupture of the membranes, spontaneous pregnancy loss <20 weeks gestation, fetal growth restriction (< 10^th ^birthweight for gestational age percentile), perinatal mortality (stillbirth or neonatal death), admission to neonatal intensive care unit, duration of stay in hospital (maternal and infant), birth weight, Apgar score at 5 minutes, and a composite neonatal morbidity indicator including respiratory distress, assisted ventilation, intraventricular haemorrhage, necrotising enterocolitis, retinopathy and pneumonia [57].

### Other maternal and infant explanatory factors

Other conditions may arise in pregnancy that influence preterm birth risk including pregnancy hypertension, gestational diabetes, antepartum haemorrhage, placental abnormalities (placenta praevia, placental abruption), congenital anomalies, onset of labour (spontaneous, induced or no labour) and mode of delivery.

### Analyses

Analyses will be by intention to treat. The primary analysis will compare the proportions of spontaneous preterm births in the two groups, using a chi-squared test. The relative risk and 95% confidence interval of preterm birth and other outcomes in women with active treatment compared with those with usual care will be calculated. In the secondary analysis of the primary outcome, logistic regression will be used to adjust for centre and any differences in important baseline characteristics. Secondary outcomes that are binary will be analysed in a similar manner, while those that are quantitative will be compared between groups using t-tests or the distribution-free equivalent, as appropriate.

Sub-group analyses will be used to explore associations between preterm birth and clotrimazole treatment by degree of *Candida *colonisation (light, moderate, heavy), *Candida albicans *and *non-albicans *species, gestational age at enrolment, and among medically indicated preterm births by indication (hypertension, growth restriction, fetal malformation, placental abnormalities). To test for differences in the effect of clotrimazole among sub-groups, a chi-squared test for interaction will be used, using the trend version for the ordered variable, degree of colonisation.

### Interim analyses: the Data Monitoring Committee

An independent Data Monitoring Committee (DMC) with established terms of reference and stopping rules will be appointed to review interim data and other evidence (including updated overviews of relevant randomised controlled trials). The DMC will:

• Advise on protocol modifications suggested by investigators or collaborators (e.g. sample size or trial endpoint modifications)

• Assess the impact and relevance of new external evidence

• Assess data quality, including completeness

• Monitor recruitment figures, balance in recruitment by centre and losses to follow-up

• Monitor evidence for treatment harm

• Monitor planned sample size assumptions

• Monitor treatment side-effects and compliance with the treatment regimen

• Recommend that the trial continues to recruit participants or that recruitment should be terminated

An interim analysis will be conducted when outcomes are known for the first 50% of women recruited. The O'Brien-Fleming method[58] will be used to preserve the overall P-value at 0.05; for the interim analysis we will test at P = 0.0031 (z = 2.95430) and for the final analysis at P = 0.0497 (z = 1.96257) (these calculations were performed using PASS).

The DMC will inform the Steering Group if in their view an arm of the study is either clearly indicated or contra-indicated. The final decision on whether to stop the trial will rest with the Steering Group.

### Ethics Approval

Ethics approval has been granted by the Northern Sydney Central Coast Area Health Human Research Ethics Committee (Protocol 0907-165M)

## Discussion

Prevention of preterm birth remains one of the most important challenges in modern maternity care. Providing *a Healthy Start to Life*, an Australian National Research Priority, is the goal of this proposal. This trial will provide definitive evidence on whether screening and treating of asymptomatic candidiasis in pregnancy significantly reduces the rate of spontaneous preterm birth. A significant reduction in preterm birth would have major resource implications with a commensurate reduction in the need for neonatal facilities, hospitalisations and longer term care for babies born preterm.

## Competing interests

The authors declare that they have no competing interests.

## Authors' contributions

JM conceived the project and all the authors contributed to design of the study. JM and CR initially drafted the protocol and all authors were involved in critical revision of the intellectual content. CR, KR and JM obtained ethics approval for the trial. CR and KR negotiated access to population health data for outcome assessment. All authors approved the final protocol.

## Pre-publication history

The pre-publication history for this paper can be accessed here:

http://www.biomedcentral.com/1471-2393/11/19/prepub
